# Increasing students’ physical activity during school physical education: rationale and protocol for the SELF-FIT cluster randomized controlled trial

**DOI:** 10.1186/s12889-017-4553-8

**Published:** 2017-07-11

**Authors:** Amy S. Ha, Chris Lonsdale, David R. Lubans, Johan Y. Y. Ng

**Affiliations:** 1 Department of Sports Science and Physical Education, The Chinese University of Hong Kong, Shatin, Hong Kong; 2 Institute for Positive Psychology and Education, Australian Catholic University, Edward Clancy Building 167-169 Albert St, Strathfield, NSW 2135 Australia; 3 Priority Research Centre for Physical Activity and Nutrition, School of Education, University of Newcastle, Callaghan, NSW 2308 Australia

**Keywords:** Physical education, Moderate-to-vigorous physical activity, School-based intervention, Health-related fitness, Self-determination theory, Basic psychological needs, Fitness dice

## Abstract

**Background:**

The Self-determined Exercise and Learning For FITness (SELF-FIT) is a multi-component school-based intervention based on tenets of self-determination theory. SELF-FIT aims to increase students’ moderate-to-vigorous physical activity (MVPA) during physical education lessons, and enhance their autonomous motivation towards fitness activities. Using a cluster randomized controlled trial, we aim to examine the effects of the intervention on students’ MVPA during school physical education.

**Methods:**

Secondary 2 students (approximately aged 14 years) from 26 classes in 26 different schools will be recruited. After baseline assessments, students will be randomized into either the experimental group or wait-list control group using a matched-pair randomization. Teachers allocated to the experimental group will attend two half-day workshops and deliver the SELF-FIT intervention for 8 weeks. The main intervention components include training teachers to teach in more need supportive ways, and conducting fitness exercises using a fitness dice with interchangeable faces. Other motivational components, such as playing music during classes, are also included. The primary outcome of the trial is students’ MVPA during PE lessons. Secondary outcomes include students’ leisure-time MVPA, perceived need support from teachers, need satisfaction, autonomous motivation towards physical education, intention to engage in physical activity, psychological well-being, and health-related fitness (cardiorespiratory and muscular fitness). Quantitative data will be analyzed using multilevel modeling approaches. Focus group interviews will also be conducted to assess students’ perceptions of the intervention.

**Discussion:**

The SELF-FIT intervention has been designed to improve students’ health and well-being by using high-intensity activities in classes delivered by teachers who have been trained to be autonomy needs supportive. If successful, scalable interventions based on SELF-FIT could be applied in physical education at large.

**Trial registration:**

The trial is registered at the Australia New Zealand Clinical Trial Registry (Trial ID: ACTRN12615000633583; date of registration: 18 June 2015).

## Background

Moderate-to-vigorous physical activity (MVPA) is associated with lower rates of chronic health issues, including diabetes and obesity in children and adolescents [1–3]. Despite the health benefits, previous research has shown that MVPA levels of adolescents are low in Hong Kong [4–6] and in other parts of the world [7, 8]. As physical activity and inactivity patterns during childhood and adolescence may be carried onto the latter stages in one’s lifespan [9, 10], increasing and sustaining adolescents’ activity levels is important. Apart from general MVPA, researchers have found that vigorous activity, independent from moderate forms, may be strongly associated with young people’s health [11]. Physical activity of vigorous intensity is difficult to accumulate during most day-to-day activities. Schools and, more specifically, physical education (PE) classes are suitable venues for promoting all forms of MVPA, which should also include vigorous forms of physical activity. This is because school PE is compulsory for school-aged students and, thus, has the potential to reach nearly all adolescents [5, 12, 13], allowing them to explore and learn activities that will ideally allow them to remain active and fit throughout their lifespans.

To increase students’ activity levels in PE, a number of school-based interventions have been designed and implemented. In a review and meta-analysis, Lonsdale et al. [14] categorized published school-based interventions into two broad types of strategies, namely teaching strategies and fitness infusion. Specifically, “teaching strategies” are methods used by teachers to maximize students’ MVPA by activity selection, class organization, and instructional practices (e.g., [15, 16]). Whereas “fitness infusion” are strategies used by teachers to supplement existing lessons with additional vigorous activities (e.g., [17, 18]). Most existing interventions have employed one of these approaches. However, as vigorous activities may lead to reduced affective responses [19, 20], fitness infusion strategies may actually negatively impact students’ motivation, and thus future participation. In response, our goal was to create an intervention using fitness infusion that could be delivering in a manner that would have a positive impact on students’ autonomous motivation for physical activity. Essentially, teachers will be trained to teach in a way that supports students’ basic psychological needs (i.e., teaching strategies). We also designed a fitness training protocol using “fitness dice” to increase students’ active participation in fitness exercises (i.e., fitness infusion), and to increase their enjoyment while doing so. The effectiveness of the intervention will be evaluated using a cluster randomized controlled trial.

### School physical education: The Hong Kong context

Physical education is a compulsory subject in Hong Kong schools. Schools are recommended to allocate 5% to 8% of total curriculum time for PE [21]. In practice, this translates to approximately 60 to 90 min of PE time per week in secondary schools. Despite being a key learning area of the Hong Kong general curriculum [21], and taught by teachers who received professional training in the subject, PE has been marginalized as it does not contribute to “academic achievements” [22]. As a result, some students are disinterested in PE and physical activity in general. Although “health and fitness” is one of the six strands of the PE key learning area [12], such aspects only take up small portions of most classes. Typically, a Hong Kong PE lesson will consist of a short warm-up period, occasionally followed by a short fitness session. Through the authors’ observation, fitness activities other than running, push-ups and sit-ups are extremely rare. Afterwards, the majority of time within the class will be spent on teaching sports skills, which focus mainly on track and field or team ball games. Students’ engagement in activities is often low, even during game play as most schools have limited playing space, therefore many students will have to sit out. In view of these issues, we designed an intervention with the aims to increase students’ involvement in fitness activities, thus their activity levels, and to make PE more fun and engaging.

The Self-determined Exercise and Learning For FITness (SELF-FIT) intervention was designed based on tenets of self-determination theory (SDT) [15, 23–25]. It is a multi-component, school-based intervention aimed to enhance teachers’ need supportiveness behaviors, students’ basic need satisfaction, motivation and their physical activity behaviors. According to tenets of SDT, three human basic psychological needs of competence (feeling competent and effective), autonomy (feel being the true origin of engaging in the activity), and relatedness (feel a sense of connection with other people) are important determinants of adaptive forms of motivation, behaviors, and well-being. When these needs are satisfied, individuals are more likely to show higher levels of adaptive motivation towards the corresponding behavior, and in turn increase their engagement in such activities. Moreover, basic need satisfaction may also directly enhance one’s psychological well-being, such as less depressive symptoms or higher quality of life [24]. Within SDT, motivation can be broadly categorized into two forms, namely autonomous and controlled motivation. Autonomous motivation represents the more adaptive form of motivation, and is represented by engaging in an activity for fun and enjoyment, or the individual feels that the outcomes of engaging in the activity would be personally important. Within the domain of school PE, this type of motivation has been found to be related to students’ MVPA during PE [26, 27] or in their leisure time [6, 27], and their psychological well-being [28, 29]. Therefore, school-based interventions designed to enhance students’ basic need satisfaction and autonomous motivation may induce positive changes in activity behaviors and psychological well-being in students.

Research has shown that students’ autonomous motivation towards PE may be affected by the interpersonal style utilized by the teacher. Students’ need satisfaction and autonomous motivation are related to teachers’ need supportive behaviors [28, 30, 31]. By definition, need supportive behaviors support individuals’ basic need satisfaction, which include provision of non-contingent positive feedback, providing choices and meaningful rationales to tasks, and taking the perspective of students and acknowledging their negative feelings [32]. Importantly, autonomy supportive teaching styles can be trained. For example, Cheon and colleagues [33] demonstrated that when teachers were trained to teach in autonomy supportive ways, students showed higher levels of autonomous motivation, greater engagement in class, and future intentions to exercise. Having higher levels of motivation may also have a carry-on effect to students’ leisure time physical activity [27]. Therefore, in the current study, we will examine whether the intervention will have an effect on students’ leisure time MVPA. Apart from teacher behaviors, we will also embed game play elements to the fitness activities. Specifically, we have designed a set of four fitness dice with interchangeable faces. Each die includes changeable exercise cards for the following aspects of health-related fitness, namely flexibility, cardiovascular fitness, upper and lower body muscular fitness (detailed descriptions are presented in the method section).

A cluster randomized controlled trial will be used to evaluate the effectiveness of the SELF-FIT intervention. Through the trial, we aim to examine whether the school-based intervention could (1) increase teachers’ need supportiveness during PE (rated by students); (2) enhance students’ basic need satisfaction and autonomous motivation towards PE, (3) increase their MVPA during school PE, and (4) increase their leisure time MVPA. Specifically, Secondary 2 (equivalent to Grade 8) students and their teachers will be recruited to take part in the trial. Participating teachers will be randomly allocated to either an experimental group or a wait-list control group after baseline measures of students are taken. Teachers in the experimental group will then attend two half-day workshops, and employ the designed intervention in their classes. We hypothesize that at follow-up, students in the experimental group, compared to those in the control group, will show higher levels of MVPA during school lessons and in their leisure time, report higher levels of perceived need support, autonomous motivation, intentions to be physically active, and psychological well-being.

## Methods

### Trial design

**Fig. 1:**
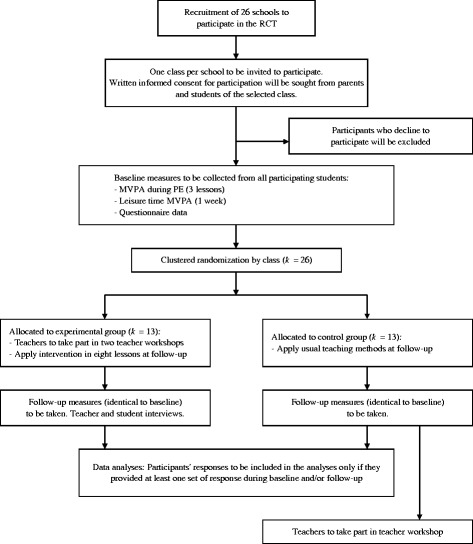
Flow diagram of the cluster randomized controlled trial

A flow diagram of our cluster randomized controlled trial protocol is shown in Fig. 1. Baseline measures, including all primary and secondary outcomes, will be taken during the first half of the school year (approximately 4 months excluding examination periods). After baseline measures are taken, schools will be randomly allocated into either an intervention or wait-list control group. Teachers allocated to the experimental group will be invited to attend two half-day teacher workshops, and will apply the intervention contents in the second half of the school year (i.e., follow-up period; approximately 4 months). By contrast, teachers in the control group will not receive any additional instructions or materials for teaching until all follow-up measures have been taken. At follow-up, all primary and secondary outcomes will be measured again.

### Sample size calculation

A sample size calculation was completed to estimate the number of schools needed for the trial. The calculation was based on our primary outcome of the percentage of time students spent in MVPA during PE lessons. Based on Lonsdale et al.’s meta-analysis [14], the expected standardized difference, or Cohen’s *d*, between intervention (using fitness infusion strategies) and control groups was 1.4. Using a more conservative estimate, we used *d* = 0.9 for our sample size calculation. Calculations were conducted using GPower 3.1.7, with an alpha level set to .05 and power to .80. The required sample size was calculated to be 42. In the current trial, each class would be treated as an individual cluster. Therefore, the calculated sample size was then multiplied by a design effect of 1 + (*m* – 1)ρ, where *m* represents the cluster size (estimated to be 30) and ρ is the intraclass coefficient (ICC). Based on a study conducted in Hong Kong [17], the ICC was estimated to be at .60. Therefore, the final number of participants required was 42 × [1 + (30-1) × .60] = 773, meaning a total of 26 schools will need to be recruited.

### Participants

To reduce the potential confounding effect of students’ age and ethnic background, we will limit recruitment to Secondary 2 (equivalent of Grade 8) students from Hong Kong schools. To recruit participating schools for the trial, invitations will be sent to 100 randomly selected secondary schools in Hong Kong (i.e., approximately 20% of all eligible schools). To reduce heterogeneity, only schools with mainly Chinese students whose mother tongue is Chinese will be invited. No other criteria were used when sending out invitations. Representatives from each of these schools will be invited to a briefing session to outline the background of the study, before they decide on their participation. Students from one Secondary 2 from each school will be invited to take part in the study. Measurements of all outcomes will only be taken from students who agree to take part in the study.

### Ethics, consent and permissions

Prior to their participation, students and their parents will provide written informed consent, and complete the Physical Activity Readiness Questionnaire [31] to ensure they are physical healthy to engage in fitness activities (i.e., no known episodes of chest pain, dizziness, or joint problems after physical activity). Specifically, information sheets regarding the study will be sent to parents via school teachers. If parents agree to their child taking part in the study after reading the information sheets, they will need to complete, sign, and return the informed consent forms to school teachers. The completed forms will then be collected by the research team from teachers. Ethical approval for the study was obtained from the Joint Chinese University of Hong Kong – New Territories East Cluster Clinical Research Ethics Committee (Ref: 2014.114).

### Randomization

Randomization will take place after baseline measures are taken. A matched-pair randomization protocol will be used. Specifically, the classes will be paired based on (1) class sex (mixed versus female versus male), (2) school type (mixed or single-sex), (3) class size (inclusive of non-participating students), (4) average family income (low versus mid versus high; to be reported by school teacher), and (5) the percentage of MVPA during PE measured at baseline. Each class will then be assigned a computer-generated random number. In each matched pair, the class with the larger assigned number will be allocated in the experimental group, while the other class will be assigned to the control group.

### Intervention

**Fig. 2:**
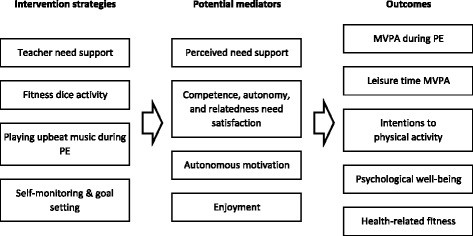
Intervention components, potential mediators, and outcomes

The SELF-FIT intervention was developed using the tenets of SDT [23]. In particular, all intervention components were focused on enhancing students’ satisfaction of the three basic psychological needs of competence, autonomy, and relatedness within the context of PE. An outline of intervention components and how these components are intended to enhance students’ basic needs satisfaction are shown in Table 1 and Fig. 2.

**Table 1:** Descriptions to intervention components and relation with potential mediators

Intervention component	Description	Potential mediators intervened
Teachers’ need support	Teachers will be trained to teach using need supportive approaches during teacher workshops. Specific behaviors we will encourage include: - Providing informational feedback - Challenge students within their capabilities - Providing meaningful choices - Providing meaningful rationale - Acknowledging students’ difficulties - Showing genuine care for students	Perceived need support, competence, autonomy, relatedness, autonomous motivation
	Teaching using the SAAFE principles: - Supportive - Active - Autonomous - Fair - Enjoyment	Perceived need support, competence, autonomy, relatedness, autonomous motivation, enjoyment
“Fitness dice”activity	Teachers will be recommended to spend 20 min of lessons using the fitness dice. - Target 4 aspects of fitness highlighted by the Education Bureau (EdB) PE curriculum – flexibility, cardiovascular fitness, muscular strength, & muscular endurance - Four customizable dice with corresponding activity cards will be provided to each teacher: 1. Flexibility; 2. Cardiovascular endurance; 3. Upper body muscles; 4. Lower body muscles. - Using either a one-to-many or circuit training format, students will complete exercises based on the toss of a fitness die. - Students could choose a level of difficulty that is optimally challenging to them. - Both individual and paired activities are included to allow students to choose from. - Teachers will invite students to provide input for the fitness activities to be included in each die.	Competence, autonomy, relatedness, autonomous motivation, enjoyment
Other motivational components	Playing music during the fitness dice activity - Students can provide input as to what music to play PE record form for self-monitoring and goal-setting - Self-monitoring - Goal-setting	Competence, autonomy, autonomous motivation, enjoyment

#### Teacher workshops

SELF-FIT intervention components will be applied using a train-the-trainer approach. That is, teachers in the experimental group will be trained to implement the intervention at their schools. Training for teachers will be provided during two four-hour teacher workshops, which is led by the lead author of this manuscript. The lead author has instructed in a university PE teaching training program for over 20 years, and has published multiple papers based on SDT. The first teacher workshop will be divided into three sections. In each of these sections, teachers will be given time to reflect upon and discuss methods relating to teaching practices. Specifically, in the first section of the workshop, teachers will be given definitions of “supportive” and “controlling” practices based on Reeves et al. [34]. Two short videos of students talking about what they like or dislike about PE teachers will be shown to highlight the importance of taking on students’ perspectives in teaching. An overview of SDT and how the three basic psychological needs are linked with student behaviors and well-being will then be provided. The second section of the workshop will be an activity session for teachers to participate in the fitness activities using the fitness dice we designed for this study (see detailed description of the fitness dice below). The practical activities will then be followed by discussions on the practicality of implementing this intervention component within schools, and potential problems teachers may face when using the dice in their lessons. Videos from a pilot study using the fitness dice in real-life settings will be shown, and teachers who implemented the intervention in the pilot study will be invited to share their experiences in using the dice. In the final section of the workshop, specific guidelines on practices to enhance students’ need for competence (e.g., providing information feedback and optimal challenges), autonomy (e.g., providing meaningful choices and rationales for instructions), and relatedness (e.g., acknowledging students’ difficulties and showing genuine care) will be provided. Furthermore, we will further present teachers with the SAAFE (Supportive, Active, Autonomous, Fair and Enjoyable) teaching principles [35–37], which were originally developed for the primary school setting, but have now been used to support the delivery of school-based physical activity interventions targeting adolescents [38–40]. The SAAFE teaching principles were designed to provide teachers with a framework for enhancing student activity levels and motivation in PE and school sport. Specifically, teachers are encouraged to: i) be *supportive* in their teaching, ii) enable their students to be *active* for the majority of the lesson, iii) provide students with *autonomy* by including elements of choice, iv) to create a lesson experience that is *fair* by providing all students with opportunity to experience success, and v) foster an *enjoyable* PE experience by focusing on fun and variety in lessons.

A second workshop will be arranged for teachers in the experimental group approximately 6 to 8 weeks after the first session. Our goal is to utilize this workshop as a reminder and booster for teachers’ need supportive behaviors. The time between the two workshops will be set so that most attending teachers will have implemented the intervention components during at least four of their lessons. Therefore, teachers returning to the workshop could present to the group new exercise ideas they may have developed, discuss barriers they might have faced during the implementation, and examine how such barriers could be overcome in school settings.

#### Fitness dice

**Fig. 3:**
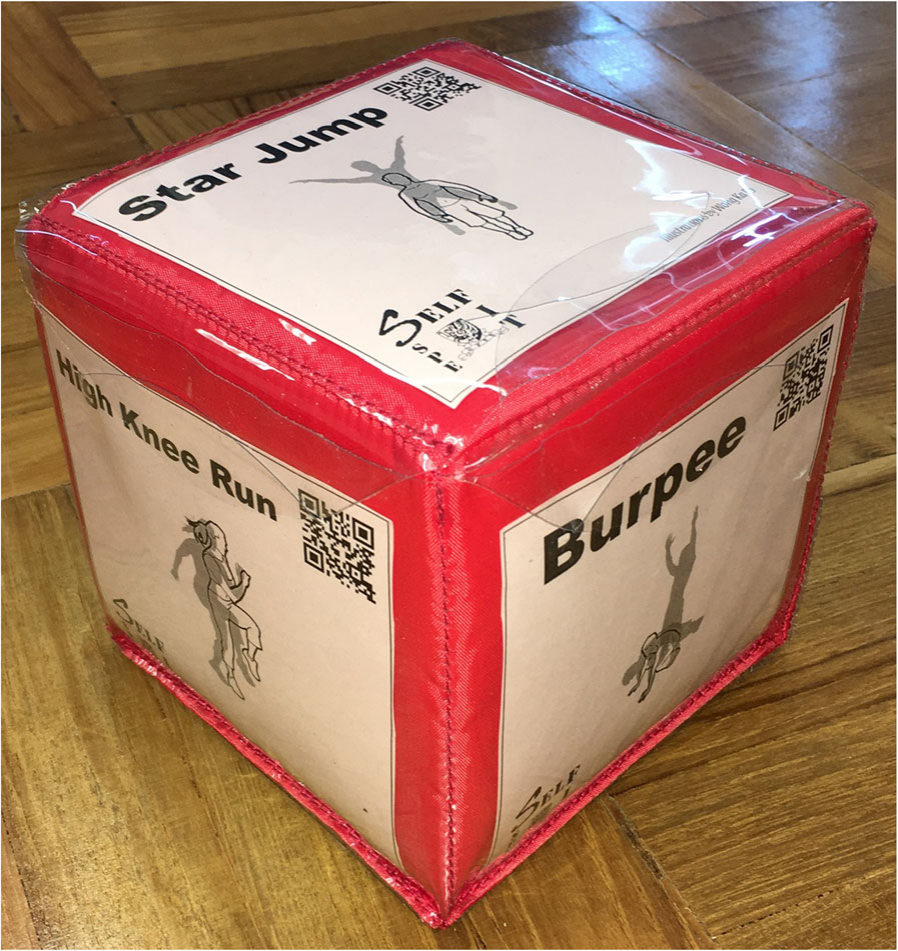
The fitness dice with interchangeable faces

One of the major components of the intervention will be the use of “fitness dice” as a fun and innovative activity to promote fitness during school PE. Our main objective is to inject an element of game-play to fitness activities, and in turn increase students’ enjoyment while engaging in such exercises. Specifically, we designed a set of dice with all six faces covered using transparent film pockets that can hold paper or other flattened contents (e.g., CD/DVD discs). Using this design, each face of a die will be interchangeable, and, therefore, can be customized to the needs of users (see Fig. 3). Put in the context of the current study, teachers will be able to insert activity cards provided by the research team and then create their own based on the i) skill and fitness levels of their students, ii) equipment available for their classes, and iii) activities favored by their students. In the long run, we will encourage teachers to invite students to choose, or to create, activities for the faces of the dice. The activity cards we initially provide teachers will be categorized into four types, namely i) flexibility, ii) cardiorespiratory fitness, iii) upper and iv) lower body muscular fitness. That is, the activities shown on the cards will aim to improve students’ flexibility (e.g., shoulder stretches), cardiovascular fitness (e.g., burpees), upper body and core muscular strength and endurance (e.g., push-ups), and lower body muscular strength and endurance (e.g., lunges), respectively. The name of the exercise, together with a graphical representation of the activity, will be printed on the activity cards. For some activities (e.g., push-ups), different difficulties of the same exercise (i.e., normal push-ups, modified push-ups) will be shown to allow students to choose the level that provide them with the optimal level of challenge. Apart from individual exercises, some exercises will involve paired work (e.g., plank hand slap). Such collaborative exercises are inserted with the aim of making the activity more interactive and fun. Quick response codes (i.e., QR Codes) linking to video tutorials, created specifically for this project, of the respective exercises are printed on the activity cards. This will provide teachers and students easy access to these videos. A DVD containing these video tutorials will also be given to teachers for reference.

Teachers will be requested to use the dice on at least eight consecutive PE lessons after they attend the workshop. This length was chosen because it allowed sufficient time for teachers and students to familiarize with the activity, but at the same time not too long to cause disruptions to teachers’ curricula. According to Lonsdale et al. [14], previous school-based PE interventions had a length of 1 to 130 weeks. In particular, fitness infusion interventions last 4 to 24 weeks. Therefore, the length of the SELF-FIT was in line with similar interventions that have been employed previously. An implementation guideline for these eight lessons will be provided to teachers. Specifically, we will recommend teachers spend 20 min using the fitness dice during each of the eight lessons. The first two lessons will be a familiarization session for both teachers and students, and we will recommend teachers use one-to-many instruction format, and utilize one die at a time. Essentially, the teacher will toss the die and instruct students to do the activity shown on the die. After the two familiarization lessons, we will recommend teachers use the dice using a circuit-training format. Essentially, depending on class size, equipment and space availability, two to four stations could be set up, and students will use a specific die for each station. Teachers could instruct students to change stations after a specific time (e.g., 1 min), so students could complete multiple circuits. We will recommend teachers increase student participation in the fitness dice activity throughout the eight-lesson period. For instance, we recommend teachers to nominate activity leaders to demonstrate the selected exercises, select activity cards to be placed in the dice, or design new exercises to be inserted. We will also suggest teachers add other game play elements, such as tossing a die for students at other stations, inserting “wild cards” in the dice faces for self-chosen activities, or adding an additional die to decide the time or repetition of the exercise to complete. Teachers will be told that they do not have to strictly follow these guidelines and they are free to improvise will the aim of encouraging maximal engagement and enjoyment of students.

A pilot study was conducted to specifically test the feasibility of using the fitness dice in a school PE setting. Two teachers from one secondary school were invited to pilot test the fitness dice in their classes (one class each; both were mixed-sex). The aim of conducting the pilot study was to collect preliminary evidence that (1) the use of the fitness dice is practically feasible in real school PE settings, and its implementation would not cause any major drawbacks or problems, (2) students found the activity more interesting compared to traditional fitness activities. To this end, we observed four lessons per class. Within these four lessons, the first two were taught using existing methods and equipment, while the fitness dice were used in the final two classes. All lessons in the pilot study were video-recorded for review purposes. Two teachers and ten students were interviewed after the pilot study. During the interviews, teachers reflected that the use of the fitness dice did not cause any major disruptions or delays in their lessons. Neither teachers nor students felt there were noticeable differences in terms of students’ intensity or volume of physical activity exerted when the fitness dice were used. Students either said they found the fitness dice activity to be more interesting than traditional fitness activities, or they felt there were no differences. No students said they preferred traditional fitness activities over using the fitness dice. The results from the pilot study suggested that the fitness dice may be a viable tool to be used for fitness activities in school PE.

#### Other motivational components

Apart from the main intervention components of need support teaching and the fitness dice, we will also recommend teachers employ other motivational components as part of the intervention. First, to encourage teachers to reflect on and improve their need supportive teaching strategies, we have designed a self-reflection questionnaire for teachers to complete after each lesson. Teachers will be asked to complete the questionnaire, designed based on the SAAFE principles, and reflect if they have adequately utilized supportive behaviors, and avoided controlling ones during that lesson. By completing the self-administered questionnaire, we hope that teachers will be reminded of the SAAFE principles and need supportive strategies, so they can use these to improve their teaching in the following lessons. Secondly, teachers will be requested to play some upbeat music during the fitness dice activity, as the introduction of music to PE may increase students’ activity levels [41]. After the familiarization lessons, teachers can also invite students to choose the music to be played in the following lessons. This may further enhance students’ perceptions of autonomy towards PE. Further, we will provide students with an activity record and goal setting form to be completed after each PE lesson. Specifically, students will be asked to record the exercises they have completed, together with the time or repetition they completed. On the form, they will also be encouraged to set goals for the week after.

### Fidelity of intervention

To ensure intervention fidelity, the content of lessons where accelerometer data are measured will be recorded by trained research assistants. Specifically, using a standardized form, research assistants will record the types of activities (e.g., stretching, ball games, track and field, fitness) during that lesson, as well as the start and end times of each set of activity. At follow-up, research assistant will also record for each activity whether the fitness dice were used and if music was played during the activity period. This will determine if some or all intervention components were implemented in lessons for the experimental group, but not in classes of the control group (i.e., intervention fidelity). At least one lesson per class will be video-recorded for review purposes. We will also ask teachers to report, after the fourth and eighth lesson during the intervention period, if they have completed the self-reflective questionnaires or allowed students to complete the record and goal-setting forms.

### Outcomes

The primary outcome of the randomized controlled trial is the percentage of time students spent in MVPA during PE classes. Secondary outcomes include percentage of time spent in MVPA during leisure time, student perceptions of need support by teachers, basic psychological need satisfaction, autonomous motivation towards PE, intention to engage in physical activity, psychological well-being, and indicators of health-related fitness. Identical measures for all outcomes will be used at both time points. The percentage of time spent in MVPA during PE will be measured using accelerometers, during three randomly selected lessons at each time point. Leisure-time MVPA will be measured once during baseline and follow-up, respectively. Questionnaire-based measures will be collected on one occasion at baseline and follow-up, respectively. Questionnaires will be administered during one of the last two PE classes of the respective time point. All accelerometer and questionnaire data will be collected at schools by research assistants who will be trained by the authors of the current manuscript. To ensure that the information record and data collection procedures will be consistent across all research assistants, they will be accompanied by the project manager during their respective first data collection sessions at schools.

#### Moderate-to-vigorous physical activity

Students’ MVPA will be measured using ActiGraph GT3X+ and wGT3X-BT accelerometers worn at the waist. For the primary outcome of MVPA during PE, measures for three lessons at each time point will be taken. Specifically, trained research assistants will administer the devices to students at the beginning of the lessons, and collect them when the lesson ends. For the secondary outcome of leisure-time MVPA, the same ActiGraph devices will be used, but an 8-day administration protocol will be applied. That is, after the devices are administered to measure students’ MVPA during PE (Day 0), instead of retrieving the accelerometers immediately after the lesson ends, they will be collected 8 days later (Day 8). That will allow data from seven full days (i.e., Days 1 to 7) to be collected if the students wear the devices correctly. Participants will be asked to wear the devices throughout their waking hours within the data collection period, except during water-based activities (e.g., swimming, bathing) or occasions where they are not allowed to wear such devices (e.g., a contact sport match). Leisure-time MVPA will only be measured once during baseline (i.e., the first semester) and follow-up (i.e., the second semester), respectively. It is worth noting that compliance to accelerometers protocols in school-based physical activity interventions targeting adolescents is typically low [42]. In the current study, accelerometer wear-time criteria will be defined as at least three weekdays with >8 h. This is consistent with previous research [43] and is associated with higher levels of compliance in school-based physical activity interventions [42].

Accelerometer-based data will be categorized as sedentary, light, moderate, or vigorous levels using the cutoff points proposed by Evenson et al. [44]. Epoch lengths of 1-s will be used to provide a more accurate representation of students’ activities [45, 46]. The percentages of time students spent in moderate and vigorous activities will be summed as a measure for MVPA. For leisure-time MVPA measures, students’ percentage of time spent in MVPA during weekdays and weekends will be used as secondary outcomes, respectively. Leisure time MVPA data will be considered valid only if students wore the devices on at least three weekdays (for weekday data), or at least one weekend day (for weekend data).

#### Perceived need support by teachers

The Learning Climate Questionnaire [47] will be used to measure students’ perceived need support from teachers (e.g., “I feel that my teacher provides me choices and options”). The short, 6-item version of the scale will be used to reduce the load on participants. English items were translated to Chinese by two individual groups of translators (two per group) using a back-translation protocol. Students will be asked to respond to the scale using a 7-point scale from 1 (*strongly disagree*) to 7 (*strongly agree*). The translated scale was administered to a group of students in a pilot study. The corresponding scale scores had a Cronbach alpha of .87.

#### Basic psychological need satisfaction

The satisfaction of basic psychological needs for competence, autonomy, and relatedness will be measured using an adapted version of the Basic Needs Satisfaction in Sport Scale [48]. The 20-item scale was originally developed to measure satisfaction of competence (e.g., “I am skilled”), autonomy (e.g., “I feel I participate in PE activities willingly”), and relatedness (e.g., “There are people who I can trust”) in a sport context, and was simultaneously developed in both English and Chinese. The Chinese version of the scale will be used. Item wordings will be adapted (only minor changes are required as the Chinese term for “sport” and “physical activity” are the same) to specify need satisfaction towards physical education. A 7-point scale from 1 (*Not true at all*) to 7 (*Very true*) will be used for responses.

#### Autonomous motivation for physical education

Students’ autonomous (eight items; e.g., “because PE is fun”) and controlled motivation (eight items; e.g., “because I’ll get into trouble if I don’t”) towards physical education will be measured using the Chinese version of the Perceived Locus of Causality Questionnaire [49]. Responses will be provided using a 5-point scale from 1 (*strongly disagree*) to 5 (*strongly agree*).

#### Intention to engage in physical activity

Students’ short- and long-term intention to engage in physical activity will be measured using two items (“Do you intend to do physical activity regularly [at least three times a week] during your leisure time in the coming week/three months?”). The Chinese translation of these items will be used. Students will be asked to select “yes” or “no” for both of these questions.

#### Psychological well-being

The Flourishing Scale [50] will be used as a pseudo-measure for students’ psychological well-being. The 8-item scale measures facets of psychological well-being including positive relationships and having meaning and purpose in life (e.g., “I am engaged and interested in my daily activities”). The Chinese translation of the scale will be used in this study. Responses will be made using a 7-point scale from 1 (*strongly disagree*) to 7 (*strongly agree*). The scale was administered to students in a pilot study, and the scale scores had a Cronbach alpha of .92.

#### Health-related fitness

Results from fitness tests used in the Hong Kong School Physical Fitness Award Scheme [51] of students will be collected and used as a measure of health-related fitness. The scheme is government-led, and therefore its test items are applied in most Hong Kong schools. Thus school teachers and students should be familiar with the standardized test items. All test items from the scheme will be used, except the skinfold measurements used as a measure for body composition, as the SELF-FIT was not specifically designed to improve such attributes of students. These include a cardiovascular fitness test (a nine-minute run protocol), one-minute curl up test, sit and reach test, and a push-up test. The test protocols can be accessed from the award scheme website [51].

### Blinding

As teachers will be required to implement the interventions in schools, they will not be blinded to group allocation. Students, however, will not be informed about the group differentiations, and therefore will be blinded to group allocation. Research assistants who are responsible for data collection will not be blinded to group allocation.

### Statistical analyses

The primary outcome to be evaluated in the trial is students’ percentage of time spent in MVPA during school PE. Secondary outcomes include percentage of time spent in leisure time MVPA, perceived need support from teachers, need satisfaction, autonomous motivation, psychological well-being, and intention to engage in physical activity. To account for the clustering nature of data, multilevel analyses will be used. Specifically, for each outcome, a three-level (time within student within class) model will be evaluated. Analyses will be conducted using MLwiN v2.26 [52]. For all outcomes, the effectiveness of the intervention will be examined through the multilevel regression equation:

$$ \mathrm{Outcome}=\mathrm{B}0+{\mathrm{Gender}}^{\ast}\mathrm{B}1+{\mathrm{Age}}^{\ast}\mathrm{B}2+{\mathrm{Group}}^{\ast}\mathrm{B}3+{\mathrm{Time}}^{\ast}\mathrm{B}4+{\mathrm{Group}}^{\ast }{\mathrm{Time}}^{\ast}\mathrm{B}5 $$

Specifically, the intervention will be considered to be effective in promoting the outcomes if the Group*Time term is positive and significant at *p* < .05.

### Teacher and student interviews

After all quantitative follow-up data are collected, qualitative interview data will be collected from teachers and students. Through individual or focus group interviews, we aim to acquire subjective reflections of teachers’ and students’ view on the intervention. More specifically, we will ask teachers and students how they compared the fitness dice activity to usual fitness activities teachers had been using. Subjective reports on changes in activity levels and teacher behaviors before and after the intervention will be sought from both teachers and students. Example questions include whether students enjoyed the fitness dice activities, whether their activity levels changed as a result of using the dice, or whether there were changes in teacher behaviors after the fitness dice were used (i.e., after the first teacher workshop). The full interview schedule will be provided upon request from the first author.

## Discussion

The SELF-FIT intervention has been designed to increase students’ MVPA during PE lessons and enhance their motivation for fitness activities. By combining approaches that are aimed to modify teacher teaching strategies and interpersonal styles, and modifications to fitness activity selection during PE, we aim to make students perceive fitness exercises to be more fun and engaging. The SELF-FIT intervention is innovative because previous PE interventions have manipulated teachers’ behaviors or infused fitness activities into existing classes, but not both. Instead, as previously suggested [17], in our intervention, modifications to teacher behavior *and* fitness infusion methods are applied, while SDT tenets where integrated into both sets of strategies. Therefore, we expect the intervention will positively impact students’ MVPA and autonomous motivation for PE.

The effects of existing school-based fitness infusion intervention may be difficult to carry over to students’ leisure time, because vigorous activity in itself may not be perceived as enjoyable [53]. However, by infusing game play elements and designing the intervention based on SDT, we aim to enhance students’ motivation for physical activity, which may in turn lead to increases in leisure time activity behaviors. For example, we will suggest that teachers implementing the intervention allow students to design their own fitness activities for the fitness dice. This may encourage students to spend additional time on fitness activities outside PE or raise their interest in these exercises. In addition, this will help students take more ownership of activities within PE. The intervention may expose students (and even teachers) to a larger variety of fitness activities, and thereby enhancing their knowledge, which may be linked to future (re-)engagement in similar activities.

Apart from physical activity behaviors, research has shown that there are secular declines in health-related fitness in adolescents across the globe (e.g., [54, 55]). As physical fitness may be related to lower rates of non-communicable diseases, this decline is alarming. Using a train-the-trainer approach, and also relatively cheap equipment (i.e., the fitness dice), the scale of the SELF-FIT intervention could be increased at a relatively low cost. Scalable interventions, such as SELF-FIT, are needed to improve adolescents’ fitness, health, and motivation for physical activity.

## Abbreviations

ICC: Intraclass coefficient
MVPA: Moderate-to-vigorous physical activity
PE: Physical education
SAAFE: Supportive, Active, Autonomous, Fair and Enjoyable
SDT: Self-determination theory
SELF-FIT: Self-determined Exercise and Learning For FITness

## References

[1] Nocon M, Hiemann T, Müller-Riemenschneider FT, Frank RS, Willich SN (2008). Association of physical activity with all-cause and cardiovascular mortality: a systematic review and meta-analysis. Eur J Cardiovasc Prev Rehabil.

[2] Sullivan PW, Morrato EH, Ghushchyan V, Wyatt HR, Hill JO (2005). Obesity, inactivity, and the prevalence of diabetes and diabetes-related cardiovascular comorbidities in the U.S., 2000–2002. Diabetes Care.

[3] Janssen I, AG LB (2010). Systematic review of the health benefits of physical activity and fitness in school-aged children and youth. Int J Behav Nutr Phys Act.

[4] Chow BC, TL MK, Louie L (2008). Children's physical activity and environmental influences during elementary school physical education. J Teach Phys Educ.

[5] Ha AS, Macdonald D, BOH P (2010). Physical activity in the lives of Hong Kong Chinese children. Sport Educ Soc.

[6] Ha AS, Ng JYY: Autonomous motivation predicts 7-day physical activity in Hong Kong students. Appl Psychol Health Well Being. 2015, 7:214-229. 10.1111/aphw.12045 25943335

[7] Hallal PC, Andersen LB, Bull FC, Guthold R, Haskell W, Ekelund U (2012). Global physical activity levels: surveillance progress, pitfalls, and prospects. Lancet.

[8] Nader PR, Bradley RH, Houts RM, SL MR, O’Brien M (2008). Moderate-to-vigorous physical activity from ages 9 to 15 years. J Am Med Assoc.

[9] Biddle SJH, Pearson N, Ross GM, Braithwaite R: Tracking of sedentary behaviours of young people: a systematic review. Prev Med. 2010, 51:345-351. 10.1016/j.ypmed.2010.07.018 20682330

[10] Telama R (2009). Tracking of physical activity from childhood to adulthood: a review. Obes Facts.

[11] Carson V, Rinaldi RL, Torrance B, Maximova K, Ball GDC, Majumdar SR, Plotnikoff RC, Veugelers P, Boule NG, Wozny P et al: Vigorous physical activity and longitudinal associations with cardiometabolic risk factors in youth. Int J Obes. 2014, 38:16-21. 10.1038/ijo.2013.135 23887061

[12] Education Bureau: An overview of the learning topics in the six strands: Physical education key learning area. In*.* Edited by Bureau E. Hong Kong: Government Logistics Department; 2013.

[13] Hills AP, Dengel DR, Lubans DR (2015). Prog Cardiovasc Dis.

[14] Lonsdale C, Rosenkranz RR, Peralta LR, Bennie A, Fahey P, Lubans DR (2013). A systematic review and meta-analysis of interventions designed to increase moderate-to-vigorous physical activity in school physical education lessons. Prev Med.

[15] Lonsdale C, Rosenkranz RR, Sanders T, Peralta L, Bennie A, Jackson B, Taylor IM, Lubans DR (2013). A cluster randomized controlled trial of strategies to increase adolescents’ physical activity and motivation in physical education: results of the motivating active learning in physical education (MALP) trial. Prev Med.

[16] Webber LS, Catellier DJ, Lytle LA, Murray DM, Pratt CA, Young DR, Elder JP, Lohman TG, Stevens J, Jobe JB (2008). Promoting physical activity in middle school girls - trial of activity for adolescent girls. Am J Prev Med.

[17] Ha AS, Lonsdale C, Ng JYY, Lubans DR (2014). A school-based rope skipping intervention for adolescents in Hong Kong: protocol of a matched-pair cluster randomized controlled trial. BMC Public Health.

[18] Scantling E, Dugdale H, Bishop P, Lackey D, Strand B (1998). The effects of two instructional formats on the heart rate intensity and skill development of physical education students. Phys Educ.

[19] Sheppard KE, Parfitt G (2008). Acute affective responses to prescribed and self-selected exercise intensities in young adolescent boys and girls. Pediatr Exerc Sci.

[20] Schneider ML, Graham DJ (2009). Personality, physical fitness, and affective response to exercise among adolescents. Med Sci Sports Exerc.

[21] Curriculum Development Council (2002). Physical education: key learning area curriculum guide (primary 1-secondary 3).

[22] Johns DP, Dimmock C (1999). The marginalization of physical education: impoverished curriculum policy and practice in Hong Kong. J Educ Policy.

[23] Ryan RM, Deci EL, Deci EL, Ryan RM (2002). Overview of self-determination theory: an organismic-dialectical perspective. Handbook of self-determination research.

[24] Ng JYY, Ntoumanis N, Thøgersen-Ntoumani C, Deci EL, Ryan RM, Duda J, Williams GC (2012). Self-determination theory applied to health contexts: a meta-analysis. Perspect Psychol Sci.

[25] Lonsdale C, Lester A, Owen KB, White RL, Moyes I, Peralta L, Kirwan M, Maeder A, Bennie A, MacMillan F (2016). An internet-supported physical activity intervention delivered in secondary schools located in low socio-economic status communities: study protocol for the activity and motivation in physical education (AMPED) cluster randomized controlled trial. BMC Public Health.

[26] Aelterman N, Vansteenkiste M, Van Keer H, Van den Berghe L, De Meyer J, Haerens L (2012). Students’ objectively measured physical activity levels and engagement as a function of between-class and between-student differences in motivation toward physical education. J Sport Exerc Psychol..

[27] Owen K, Smith J, Lubans DR, Ng JYY, Lonsdale C (2014). Self-determined motivation and physical activity in children and adolescents: a systematic review and meta-analysis. Prev Med.

[28] Standage M, Gillison FB, Ntoumanis N, Treasure DC (2012). Predicting students’ physical activity and health-related well-being: a prospective cross-domain investigation of motivation across school physical education and exercise settings. J Sport Exerc Psychol..

[29] Lubans DR, Smith JJ, Morgan PJ, Beauchamp MR, Miller A, Lonsdale C, Parker P, Dally K (2016). Mediators of psychological well-being in adolescent boys. J Adolesc Health.

[30] Taylor IM, Ntoumanis N (2007). Teacher motivational strategies and student self-determination in physical education. J Educ Psychol.

[31] Sparks C, Dimmock J, Lonsdale C, Jackson B (2016). Modeling indicators and outcomes of students’ perceived teacher relatedness support in high school physical education. Psychol Sport Exerc.

[32] Deci EL, Ryan RM (1987). The support of autonomy and the control of behavior. J Pers Soc Psychol.

[33] Cheon SH, Reeve J, Moon IS (2012). Experimentally based, longitudinally designed, teacher-focused intervention to help physical education teachers be more autonomy supportive toward their students. J Sport Exerc Psychol.

[34] Reeve J (2009). Why teachers adopt a controlling motivating style toward students and how they can become more autonomy supportive. Educ Psychol.

[35] Lubans DR, Morgan PJ, Weaver K, Callister R, Dewar DL, Costigan SA, Finn TL, Smith J, Upton L, Plotnikoff RC (2012). Rationale and study protocol for the supporting children’s outcomes using rewards, exercise and skills (SCORES) group randomized controlled trial: a physical activity and fundamental movement skills intervention for primary schools in low-income communities. BMC Public Health.

[36] Cohen KE, Morgan PJ, Plotnikoff RC, Callister R, Lubans DR (2015). Physical activity and skills intervention: SCORES cluster randomized controlled trial. Med Sci Sports Exerc.

[37] Lonsdale C, Sanders T, Cohen KE, Parker P, Noetel M, Hartwig T, Vasoncellos D, Kirwan M, Morgan P, Salmon J (2016). Scaling-up an efficacious school-based physical activity intervention: study protocol for the ‘Internet-based professional learning to help teachers support activity in Youth’ (iPLAY) cluster randomized controlled trial and scale-up implementation evaluation. BMC Public Health.

[38] Lubans DR, Smith JJ, Peralta LR, Plotnikoff RC, Okely AD, Salmon J, Eather N, Dewar DL, Kennedy S, Lonsdale C (2016). A school-based intervention incorporating smartphone technology to improve health-related fitness among adolescents: rationale and study protocol for the NEAT and ATLAS 2.0 cluster randomised controlled trial and dissemination study. BMJ Open.

[39] Costigan SA, Eather N, Plotnikoff RC, Taaffe DR, Pollock E, Kennedy SG, Lubans DR (2015). Preliminary efficacy and feasibility of embedding high intensity interval training into the school day: a pilot randomized controlled trial. Prev Med Rep.

[40] Smith JJ, Morgan PJ, Plotnikoff RC, Dally KA, Salmon J, Okely AD, Finn TL, Lubans DR (2014). Smart-phone obesity prevention trial for adolescent boys in low-income communities: the ATLAS RCT. Pediatrics.

[41] Barney D, Prusak KA (2015). Effects of music on physical activity rates of elementary physical education students. Phys Educ.

[42] Borde R, Smith JJ, Sutherland R, Nathan N, Lubans DR (2017). Methodological considerations and impact of school-based interventions on objectively measured physical activity in adolescents: a systematic review and meta-analysis. Obes Rev.

[43] Cain KL, Sallis JF, Conway TL, Van Dyck D, Calhoon L (2013). Using accelerometers in youth physical activity studies: a review of methods. J Phys Act Health.

[44] Evenson KR, Catellier DJ, Gill K, Ondrak KS, McMurray RG (2006). Calibration of two objective measures of physical activity for children. J Sports Sci.

[45] Aibar A, Chanal J (2015). Physical education: the effect of epoch lengths on children’s physical activity in a structured context. PLoS One.

[46] Sanders T, Cliff DP, Lonsdale C (2014). Measuring adolescent boys’ physical activity: bout length and the influence of accelerometer epoch length. PLoS One.

[47] Williams GC, Wiener MW, Markakis KM, Reeve J, Deci EL (1994). Medical students’ motivation for internal medicine. J Gen Intern Med.

[48] Ng JYY, Lonsdale C, Hodge K (2011). The basic needs satisfaction in sport scale (BNSSS): instrument development and initial validity evidence. Psychol Sport Exerc.

[49] Lonsdale C, Sabiston CM, Taylor IM, Ntoumanis N (2011). Measuring student motivation for physical education: examining the psychometric properties of the perceived locus of causality questionnaire and the situational motivation scale. Psychol Sport Exerc.

[50] Diener E, Wirtz D, Tov W, Kim-Prieto C, Choi D-w, Oishi S, Biswas-Diener R (2010). New well-being measures: short scales to assess flourishing and positive and negative feelings. Soc Indic Res.

[51] Physical Education - School Physical Fitness Award Scheme. http://www.edb.gov.hk/en/curriculum-development/kla/pe/references_resource/spfas/. Accessed 19 July 2016.

[52] Rasbash J, Steele F, Browne WJ, Gostein H (2012). A user’s guide to MLwiN, v2.26: Centre for Multilevel Modelling, University of Bristol.

[53] Ekkekakis P, Parfitt G, Petruzzello SJ (2011). The pleasure and displeasure people feel when they exercise at different intensities. Sports Med.

[54] Cohen DD, Voss C, Taylor MJD, Delextrat A, Ogunleye AA, Sandercock GRH (2011). Ten-year secular changes in muscular fitness in English children. Acta Paediatr.

[55] Moliner-Urdiales D, Ruiz JR, Ortega FB, Jiménez-Pavón D, Vicente-Rodriguez G, Rey-López JP, Martínez-Gómez D, Casajús JA, Mesana MI, Marcos A (2010). Secular trends in health-related physical fitness in Spanish adolescents: the AVENA and HELENA studies. J Sci Med Sport.

